# Surveillance of adverse drug events associated with etanercept prescribed for juvenile idiopathic arthritis in a single center up to 9-years: A retrospective observational study

**DOI:** 10.1371/journal.pone.0204573

**Published:** 2018-11-09

**Authors:** Jeong Yun Choi, Jee Eun Chung, Ji Hyun Park, Yoon Sook Cho, Yong Woo Jung, Soo An Choi

**Affiliations:** 1 Department of Pharmacy, Seoul National University Hospital, Seoul, South Korea; 2 College of Pharmacy, Hanyang University, Ansan, South Korea; 3 College of Pharmacy, Korea University, Sejong, South Korea; IRCCS E. Medea, ITALY

## Abstract

The introduction of biologic agents opened a new era of treatment of juvenile idiopathic arthritis (JIA) over the past decade. From clinical experience, it appears that biological agents are well tolerated overall, and serious adverse events are rare. However, such clinical studies have not been conducted in Korea. Therefore, we examined the safety profile of JIA patients with biologics in a single center in Korea. All JIA outpatients treated from April 2004 to June 2013 were enrolled and retrospectively reviewed. Pharmacy-based surveillance of adverse drug events (ADEs) was identified by recording the patient's symptoms in the medical record and suspected ADEs were additionally explored by screening laboratory test values and observing changes in medication orders. Finally, 83 patients were enrolled and experienced 109 ADEs in 52 patients. Most ADEs (99.1%) were mild to moderate in severity assessment. The total follow-up time was 328 patient-treatment years and the overall rate of ADEs was 0.33 per patient-years for etanercept. Infection including upper respiratory tract was the most common ADE and concomitant corticosteroids contributed to the risk of infections. If the dose of prednisolone increases 0.34 mg/kg/day, the probability of developing infections increases 3.29 times. Also, all 11 patients who stopped etanercept with injection site reactions were receiving a single use prefilled syringe. In our study, etanercept appears well tolerated and safe. Children affected by JIA should be carefully monitoring so as to limit the risk of ADEs during etanercept as much as possible. To gain further knowledge about risk profiles, national collaboration for the accumulation of long-term data should be encouraged in Korea.

## Introduction

Juvenile idiopathic arthritis (JIA) is not a single disease, but a heterogeneous group of diseases characterized by arthritis of unknown etiology with onset before the age of 16 years and lasts more than 6 weeks [1]. JIA is one of the most common chronic diseases of children, with a prevalence of approximately 19.8 cases per 100,000 [2]. Most patients with JIA continue to have the active disease even after 10 years of onset, leading to disease progression into adulthood in many patients [3]. Joint damage can lead to physical disability in many affected children if proper treatment is not provided. Additional morbidity associated with JIA can be due to its treatment or effects on growth and development [4].

Tumor necrosis factor (TNF) is thought to play an important role in the pathogenesis of JIA, and high TNF levels have been found in both serum and synovial fluid of children with JIA [5]. The serum level of soluble TNF receptors has been demonstrated to correlate with disease activity. Therefore, with the advent of TNF antagonists, the treatment options for patients with rheumatic diseases have been greatly improved. In addition, biologic agents (BAs), including anti-TNF therapies, have been shown to be highly effective in treating JIA patients who were unresponsive to traditional therapies [6].

These BAs have provided exciting new therapeutic options in the last decade. Currently, TNF-α inhibitors are recommended by the American College of Rheumatology (ACR) for the treatment of JIA refractory to standard therapy [7]. Etanercept (approved since 2000) is the most commonly prescribed TNF-α inhibitor for polyarticular JIA. However, biologics decrease a patient’s immune response. In addition, infections are a concern in patients treated with anti-TNF-α agents and the long-term side effects of controlling the immune system are not yet fully understood [8]. With the increasing use of biologics worldwide, rare serious adverse events (SAEs) (infections, malignancies, heart failure, demyelinating disorders, lupus-like disease) have emerged [8–11].

Since Lovell et al first reported the use of etanercept in 69 patients with JIA in a controlled clinical trial [12], several other open label and registry studies were performed in Europe and the USA [13–17]. Most studies have focused on efficacy and long-term safety, in particular SAEs. From clinical experience, it appears that BAs are well tolerated overall, SAEs are rare, and their risk-benefit profile strongly favors benefit [13, 15]. However, such clinical studies have not been conducted in Korea. Therefore, we examined the safety profile up to 9 years treatment of active JIA patients with biologics in a single center in Korea. In addition, we investigated the association between the adverse drug events (ADEs) of etanercept and various influence factors, such as disease activity or concomitant medications.

## Material and methods

### Data collection

All JIA outpatients treated with biologics (etanercept, infliximab, adalimumab, abatacept, and anakinra) from April 2004 to June 2013 at Seoul National University Children’s Hospital (a 315-bed, tertiary care academic medical center in South Korea) were enrolled and retrospectively reviewed. Data from patients who reached the age of 19 were excluded from the safety analysis. This takes into account changes in the medical services and biologics in accordance with the pediatric age criteria in Korea. The insurance criteria of etanercept in Korea should be satisfied with treatment history of conventional DMARDs and active polyarticular JIA in patients 2~18 years of age. Therefore, they had active disease under ongoing therapy despite treatment with non-steroidal anti-inflammatory drugs (NSAIDs) and disease modifying antirheumatic drugs (DMARDs), including methotrexate (MTX), at baseline and added to or changed biologics.

Data for the study, including determination of eligibility criteria, were obtained from review of the electronic medical records (EMR) from the clinics, clinic visit notes, and linked patient data from the pharmacy database. The collected data were as follows: (1) demographic data (age, sex, weight, height, and JIA duration); (2) laboratory data [human lymphocyte antigen (HLA)-B27, fluorescent antinuclear antibody (FANA), liver enzymes, and complete blood count (CBC)]; (3) biologic medication history (dosage, administration information of BAs, status of concomitant medications—anti-inflammatory or immunosuppressants or DMARDs); (4) ADEs data (signs/symptoms—system or organ affected, onset, handling means—discontinuation or dose reduction, improvement due to biologics interruption, causality, and severity).

The etanercept dose was either 0.4 mg/kg twice a week (maximum dose 25 mg per injection) or 0.8 mg/kg once a week (maximum dose of 50 mg/week). Etanercept is supplied as a single-use prefilled syringe (25 and 50 mg) or a lyophilized powder (25 mg) for reconstitution. NSAIDs and corticosteroids, if needed, and MTX or other previous DMARDs, if tolerated, were continued.

The study was conducted in accordance with the Declaration of Helsinki and its amendments and was approved by the Institutional Review Board (IRB) of Seoul National University Hospital (1305635492) [18]. We submitted the waiver of informed consent for data collection and analyses according to Human Research Protection Program (HRPP) based on 45 CFR 46 subpart A 46.116. Patients’ signed consents were not obtained. However, our study was approved by IRB. IRB imposes a strict limitation on retrospective data collection without signed consent. Consequently, we were able to see only their clinical data, but not individual information. The entire process of retrospective data collection was acceptable only after the representative researcher submitted a written declaration.

### Identification and classification of ADEs

The definition of ADEs applied to our study followed that of the World Health Organization (WHO) [19]. Pharmacy-based surveillance of ADEs was identified by recording the patient's symptoms in the medical record and when the physician recognized it or suspected it as ADEs. Suspected ADEs were additionally explored by screening laboratory test values and observing changes in medication orders. We included in the analysis only if there is no difference between their report and the additional investigation. Clinical manifestations of the adverse events were classified using the WHO-adverse reaction terminology (ART) system. The causality of a drug for ADEs was assessed using the WHO-Uppsala Monitoring Centre (WHO-UMC) criteria, which comprised six categories: certain, probable, possible, unlikely, conditional, and unassessable [20]. Causality was independently assessed by two trained pharmacists. Serious ADEs were defined as events that were fatal or life-threatening, required hospitalization or prolonged an existing hospitalization, resulted in a persistent or significant disability or incapacity, or resulted in a congenital anomaly or birth defect [21]. In our study, the Hartwig’s Severity Assessment Scale was applied to evaluate the severity of ADEs [22].

For rates of events per patient-year of therapy, the duration of exposure to etanercept was calculated for all patients from the beginning of that. Statistical analysis was performed using Microsoft Excel 2013 and the Statistical Package for Social Sciences 22 (SPSS, Inc., Chicago, IL, USA). The association of influence factors for ADEs was analyzed using logistic regression analysis, with the dependent variable being the presence or absence of ADEs. These included the age at which biologics were initiated, sex, dosing period, and presence or absence of corticosteroids. In addition, dosage of biologics and MTX and number of concomitant DMARD as related factor were assessed for each individual ADE to reflect changes in body weight and treatment regimen. Univariable and multivariable analyses were performed. The odds ratio (OR) and 95% confidence interval (CI) were calculated for all influence factors to ADEs.

## Results

### Patient characteristics

**Fig 1:**
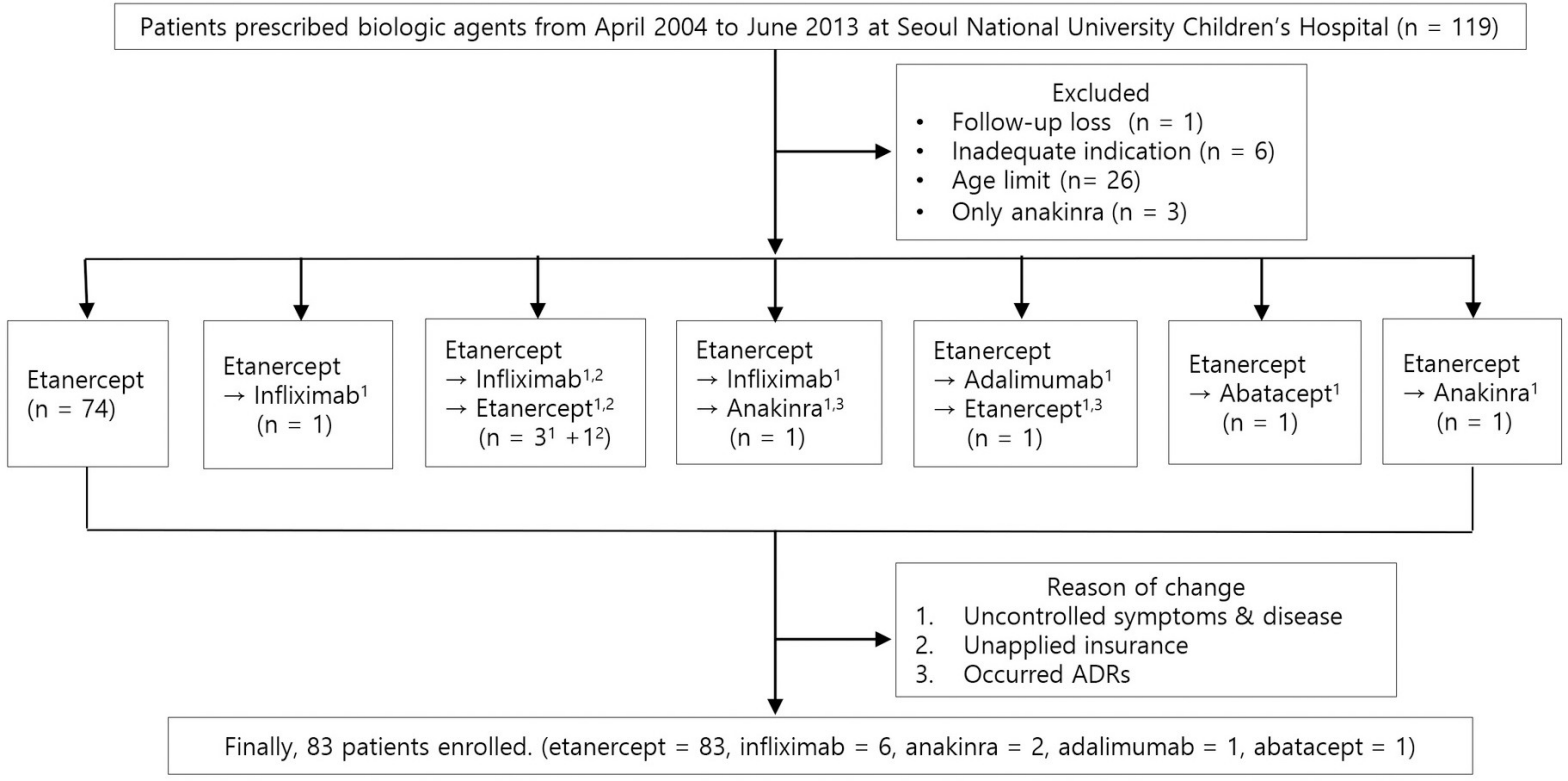
Flowchart of patients enrolled in the study using biologic agents. →;first change, ⇒; second change.

All patients were treated by experienced pediatric rheumatologists. Seventy-seven patients used only one biologic agent during the observation period: 74 were on etanercept and 3 on anakinra. Nine patients switched to a second or third agent owing to uncontrolled symptoms or disease, unapplied insurance, and occurrence of ADEs (Fig 1). Table 1 shows the demographic and JIA therapy characteristics of patients included in the study. Patients with active polyarticular-course JIA were enrolled in this study. Twenty-seven patients (31%) were HLA B-27 positive and 23 patients (27%) were FANA positive. Most patients received etanercept, and the mean ± SD age of the patients at diagnosis ranged from 10.5 ± 4.5 years to 15 ± 4.7 years in 2013. The patients who had taken etanercept had JIA duration of 87 months and dosing period of 46.3 months. All six patients who were on infliximab experienced etanercept exposure and just 8.2 months of dosing period. The ADEs maximum observation period was 9 years, 3 years and 2 years at etanercept, infliximab, and anakinra, respectively. In total, 54 patients experienced 122 ADEs. The physicians decided to discontinue biologics in 30 events and subsequently, 19 (63%) events improved.

**Table 1:** Patient demographics and characteristics of JIA therapy.

	Abatacept	Adalimumab	Anakinra	Etanercept	Infliximab
Patients/ female	1/0	1/1	5/2	83/44	6/3
Age (mean±SD, range)					
At 2013 years	11	11	8.4±5.1 (0.83~17)	15±4.7 (5~25)	17.9±2.5 (14~22)
At diagnosis	11	11	7.1±4.8 (0.5~16)	10.5±4.5 (1~18)	13.7±3.1 (8~18)
JIA duration^a^^,^ ^b^ (months)	39.8	42.2	69.8 (3.4~160.3)	87 (5.1~184.2)	127.5 (77.0~165.0)
Dosing period^a^ (months) (range)	0.5	2	12.1 (3.4~24.7)	46.3 (1.8~110.7)	8.2 (0.5~36.9)
HLA B-27/FANA positive	-/-	-/1	1/-	26/23	1/2
Adverse drug events					
Total (patients/events)	-	-	3/5	52/109	5/8
DC biologics (events)	-	-	-	22	8
→ Improved ADEs	-	-	-	14	5
Concomitant use of DMARDs^c^ (%)					
None	-	-	2 (40)	6 (7.2)	-
MTX	1 (100)	1 (100)	2 (40)	57 (68.7)	4 (66.6)
MTX + SSLZ	-	-	-	17 (20.5)	1 (16.7)
MTX + HCQ	-	-	-	3 (3.6)	-
MTX + SSLZ + HCQ	-	-	-	-	1 (16.7)
CPM	-	-	1 (20)	-	-
Concomitant use of MTX (%)					
No of patients	1 (100)	1 (100)	2 (40)	77 (92.8)	6 (100)
Dose^a^ (mg/kg/day)	0.29	0.34	0.36	0.26	0.25
Concomitant use of steroids (PD/DFZ)					
No of patients	-	-	1/-	32/1	1/1
Dose^a^ (mg/kg/day)	-	-	0.31/-	0.34/0.05	0.12/0.15
Concomitant use of NSAIDs					
No of patients	1	1	2	51	5
	Piroxicam	Piroxicam	Celecoxib	Tenoxicam(25), celecoxib(19), meloxicam(18), piroxicam(20), naproxen(4)	Celecoxib, tenoxicam, meloxicam(3),

^a^mean value (range)

^b^from the age at diagnosis to June 2013

^c^included duplicate cases

JIA, juvenile idiopathic arthritis; SD, standard deviation; DC, discontinued; DMARDS, disease modifying anti-rheumatic drugs; MTX, methotrexate; HCQ, hydroxychloroquine; CPM, cyclophosphamide; SSLZ, sulfasalazine; PD, prednisolone; DFZ, deflazacort; NSAIDs, non-steroidal anti-inflammatory drugs

Over the study period, 80 (93%) of the 86 patients received DMARDs concomitantly, and MTX was the most commonly used DMARD. Moreover, 36 (42%) and 77 (90%) patients received corticosteroids and NSAIDs, respectively.

### Assessment of ADEs and risk factors

Each patient who received abatacept and adalimumab did not report any ADEs, and AE analysis was not performed. In addition, the number of patients treated with anakinra and infliximab was very small, and this was not included in the analysis of ADEs due to the probability of underreporting. The severity of ADEs in etanercept was assessed with Hartwig’s scale and the following results were observed: 54 events were mild (49.5%), 54 events were moderate (49.5%), and one event was severe (0.9%). In the causality assessment based on WHO-UMC criteria, the number of “certain” cases was 20 (18.3%) for etanercept. All ADEs, causality and severity of ADEs are listed in Table 2. Etanercept was discontinued in 22 cases and withdrawn in 11 patients who received a single use prefilled syringe owing to injection site reaction (ISR). The total follow-up time was 328 patient-treatment years and the overall rate of ADEs was 0.33 per patient-years (Table 3). The major ADEs were infection (42), neuropsychiatric symptoms (22), ISR (17), generalized skin reaction (7), and uveitis (5).

**Table 2:** Causality and severity assessment of ADEs in etanercept.

	**No. of ADEs** **(%)**	**No. of drop-out (%)**	**Casualty assessment**				**Severity assessment**		
			Certain	Probable	Possible	Unlikely	Mild	Moderate	Severe
**Neuropsychiatric symptom**									
** Headache**	13 (11.9)		1		12		9	4	
** Numbness**	2 (1.8)			2			2		
** Seizure**	1 (0.9)				1			1	
** Tremor**	1 (0.9)				1		1		
** Fatigue**	1 (0.9)				1		1		
** Syncope**	2 (1.8)	1 (4.5)			2			2	
** Hearing impairment**	2 (1.8)				2		1	1	
**Infection**									
** URI**	36 (33.0)	3 (13.6)			36		8	28	
** Pneumonia**	3 (2.8)	1 (4.5)			3			2	1
** Chicken pox**	1 (0.9)	1 (4.5)			1			1	
** Herpes zoster**	2 (1.8)	1 (4.5)			2		1	1	
**Uveitis**	5 (4.6)			3	2		1	4	
**Injection site reaction**	17 (15.6)	11* (50.0)	17				17		
**Generalized skin reaction**	7 (6.4)	2 (9.0)	1	1	4	1	3	4	
**GI symptom**	5 (4.6)	1 (4.5)			5		3	2	
**Elevated hepatic**	2 (1.8)				2		1	1	
** Enzyme**		1 (4.5)							
**Bleeding**	2 (1.8)				2		1	1	
**Fever**	6 (5.5)		1		5		4	2	
**Dyspnea**	1 (0.9)			1			1		
**Total (%)**	109	22 (20.2)	20 (18.3)	7 (6.4)	81 (74.3)	1 (0.9)	54 (49.5)	54 (49.5)	1 (0.9)

*Single use prefilled syringe; URI, upper respiratory infection; GI, gastrointestinal.

**Table 3:** Adverse drug events of etanercept.

	**Etanercept (328 Patient treatment years)**		
	No of ADEs (%)	No of patients (%)	No of ADEs/ 100 patient-years
**Infection**	**42 (38.5)**	**30 (36.1)**	**12.8**
** Upper respiratory infection**	36 (33)	28 (33.7)	11.0
** Pneumonia**	3 (2.8)	2 (2.4)	0.9
** Chicken pox**	1 (0.9)	1 (1.2)	0.3
** Herpes zoster**	2 (1.8)	2 (1.2)	0.6
**Injection site reaction**	**17 (15.6)**	**17 (20.5)**	**5.2**
**Neuropsychiatric symptom**	**22 (20.2)**	**22 (26.5)**	**6.7**
** Headache**	13 (11.9)	13 (15.7)	4.0
** Numbness**	2 (1.8)	2 (2.4)	0.6
** Hearing impairment**	2 (1.8)	2 (2.4)	0.6
** Seizure**	1 (0.9)	1 (1.2)	0.3
** Tremor**	1 (0.9)	1 (1.2)	0.3
** Fatigue**	1 (0.9)	1 (1.2)	0.3
** Syncope**	2 (1.8)	2 (2.4)	0.6
**Generalized skin reaction**	**7 (6.4)**	**7 (8.4)**	**2.1**
**Infusion reaction**	**-**	**-**	**-**
**Uveitis**	**5 (4.6)**	**5 (6.0)**	**1.5**
**Bleeding**	**2 (1.8)**	**2 (2.4)**	**0.6**
**Fever**	**6 (5.5)**	**6 (7.2)**	**1.8**
**GI symptom**	**5 (4.6)**	**5 (6.0)**	**1.5**
**Dyspnea**	**1 (0.9)**	**1 (1.2)**	**0.3**
**Elevated hepatic enzyme**	**2 (1.8)**	**2 (2.3)**	**0.6**
**Total**	**109**	**52 (62.6)**	**33.1**

We analyzed the association between influence factors and the more frequent ADEs, infection, and neuropsychiatric symptoms of etanercept further. ADEs related to infection had significant variables such as age, sex, dosing duration, and concomitant steroid dosage (Table 4). A final multivariable analysis showed that etanercept exposure duration and steroid dose significantly correlated with ADEs related to infection. If the dose of prednisolone increases 0.34 mg/kg/day, the probability of developing infections increases 3.29 times (p = 0.022). Neuropsychiatric ADEs of etanercept was not associated with a few influence factors.

**Table 4:** Association between influence factors and frequent ADEs in etanercept.

	Infection					Neuropsychiatric ADEs		
Characteristics	No of patients	Univariable analysis		Multivariable analysis		No of patients	Univariate analysis	
		OR (95% CI)	P Value	OR (95% CI)	P Value		OR (95% CI)	P value
Age (start), years		0.9 (0.832–0.974)	0.009				1.065 (0.959–1.182)	0.239
Sex								
Female	27	1 [Reference]				12	1 [Reference]	
Male	3	0.248 (0.100–0.614)	0.003	0.402 (0.141–1.148)	0.089	5	1.010 (0.325–2.896)	0.986
DMARDs, No								
0	4	1 [Reference]				3	1 [Reference]	
1	8	0.929 (0.319–2.700)	0.892			8	0.725 (0.178–2.956)	0.653
2	18	1.083 (0.309–3.802)	0.901			6	1.667 (0.355–7.821)	0.517
Duration, days		1.001 (1.000–1.001)	0.001	1.001 (1.000–1.001)	0.022		1.000 (0.999–1.000)	0.462
Methotrexate								
Dose (mg/kg/wk)		0.554 (0.056–5.446)	0.612				0.224 (0.007–7.476)	0.404
Etanercept								
Dose (mg/kg/wk)		1.216 (0.753–1.936)	0.424				0.638 (0.251–1.619)	0.344
≤0.4 mg						11	1 [Reference]	
>0.4 mg						6	0.851 (0.316–2.291)	0.750
Steroid								
Not concomitant	16	1 [Reference]				9	1 [Reference]	
Concomitant	14	2.033 (0.971–4.257)	0.060			8	1.339 (0.501–3.581)	0.561
Dose (mg/kg/day)		7.786 (1.464–41.399)	0.016	9.674 (1.396–67.026)	0.022		0.531 (0.056–5.018)	0.580

OR, odds ratio; CI, confident interval; wk, week

## Discussion

The introduction of BAs opened a new era of treatment of JIA over the past decade. Data on long-term efficacy and safety are now available and are of great importance. Extension studies of original clinical trials and national registries were performed in Europe or North America [12, 14–17, 23]. However, until recently, data from other long-term studies were not available in Korea. We have had experience with the use of biologics in JIA treatment since 2002 and performed ADE surveillance in all pediatric patients who received BAs for the past 9 years. To the best of our knowledge, our retrospective study on the safety of biologics, such as etanercept, in JIA patients is the longest study conducted to date in Korea.

Similar to other studies, the tolerability of etanercept in JIA was overall good in our study [24–26]. In the 328 patient-treatment years of our study, 109 ADEs were recorded. The most common ADEs were infections of the upper respiratory tract, neuropsychiatric symptoms, and ISRs. In our surveillance, 62.6% of ADEs were observed and usually not serious. All ADEs were treated, but led to the discontinuation of therapy in 26.5% (22/83) of the patients. Long-term prospective study by Gerloni et al showed one or more ADEs in 57.7% of treatments. The TREAT (Trial of early aggressive therapy) study reported 51 infections, and 58 (45.7%) patients reported an infection (19.8/100 patient-years) in CLIPPER study [27, 28]. In our surveillance, 30 patients (36.1%, 12.8/100 patient-years) reported an infection, and upper respiratory infections were the most frequent ADE similar to other studies [26].

In the long-term open-label study, the rate of ADEs, SAEs, and serious infections was low even after 4–8 years of continuous treatment with etanercept [13, 14]. However, the serious infection rate in registries seems to be higher in patients receiving biologics compared to patients treated with MTX. In our study, discontinuation of etanercept owing to infections was reported in six patients. Among them, complications such as severe pneumonia and herpes zoster led to hospitalization and subsequent discontinuation of the drug. The occurrence and aggravation of infections is a major concern for patients with immune disorders and probably more important if simultaneous treatment with corticosteroids, conventional immunosuppressants, and biologics is performed. Among JIA patients, the infection rates did not increase with MTX or TNF-inhibitor use, but was significantly increased with at least a moderate dose of glucocorticoids. The risk of infection was three-fold higher in patients exposed to glucocorticoid doses >10 mg/day [11]. This result is in agreement with the results of our analysis, influencing the association between prednisolone dose and chance to develop infections. Also, above mentioned (Table 4), total etanercept treatment duration is significantly correlated with ADEs related to infection. Our surveillance is dependent on chart audit for ADE detection. Therefore, we think that this association is due to the fact that the longer the treatment period, the more often it is observed in the outpatient clinic. Actually, the mean duration of JIA for patients reporting infection during etanercept was longer (108 months, range 19.8–184.2) than total etanercept group experienced ADEs. Opportunistic infection such as tuberculosis is another major concern associated with TNF-α blockers due to their potential pro-infective action. Because Korea has the highest prevalence of tuberculosis among OECD countries, this issue is very important to us [29]. Once etanercept treatment was decided, all patients were screened for tuberculosis as the recommendation of guideline [7]. Therefore, we did not experience any cases of newly developed tuberculosis and reactivation of latent tuberculosis during etanercept therapy.

In addition, we observed a high frequency of neuropsychiatric symptoms (26.5%). The neuropsychiatric manifestations with etanercept were headache, numbness, seizure, tremor, fatigue, syncope, and hearing impairment. Unlike previous studies [12, 17, 30], severe unusual aggressiveness, central nervous system demyelination, and optic neuropathy were not reported. The cases of unusual aggressiveness, severe headache and pain amplification syndrome were clearly dose dependent according to the report (occurring in patients not responders to the conventional dose or treated with higher doses) and disappeared with suspension of treatment or with dosage reduction to 0.4 mg/kg [24]. However, as shown in Table 4, there was no significant relationship between etanercept dose and neuropsychiatric ADEs. In our study, only one patient who experienced syncope discontinued etanercept and others did not need any intervention.

Transient ISRs were described in around 39% of patients with JIA who were treated with etanercept [12]. In our study, 17 patients (20.5%) manifested injection site swelling, itching, and pain that subsequently led to the withdrawal of the drug in 11 of them. Improper injection techniques associated with drugs or excipients, including stimulation or immune-mediated inflammatory processes, can cause ISR [31]. Foreign proteins may cause direct or indirect inflammatory responses. Therefore, it is not surprising that ISRs are by far the most common side effect associated with etanercept. Patients with ISRs generally responded well to the treatment and therapy interruption was not required. The lesions resolved within 2–3 days. Anti-drug antibodies (ADAs) to etanercept as analyzed in the CLIPPER trial were rarely detectable and mostly inconsistent. The presence of etanercept antibodies did not have an apparent impact on efficacy or safety [27, 32]. The histology of etanercept ISRs is consistent with delayed-type hypersensitivity reaction [33]. Significant and severe ISRs are rare in etanercept regardless of its dose. Unlike previous studies, the most common feature of ISRs observed in our study was injection site pain. Among the 20 patients who received a single use prefilled syringe 11 (55%) patients discontinued the drug owing to ISRs. All the patients discontinued the single use prefilled syringe and switched to vials, and 10 of them showed improved ISRs. None of the studies has explored the exact reason for the frequency difference between the type of formulation and ISRs. We estimated that it caused differences in pH and osmotic pressure owing to differences in additives of the two formulations and the time required for injection after refrigerated storage[34].

Uveitis is a major clinical complication that limits the extraarticular quality of life of JIA. In clinical trials, uveitis is rarely reported as an AE, while in the BIKER registry, there were 78 cases of uveitis as an AE following etanercept treatment, 37 cases following adalimumab, and 49 cases after MTX treatment [35]. The well-known risk factors for uveitis include younger age, ANA positivity, and the JIA category of oligoarticular JIA [36, 37]. According to a recent study, patients with a history of uveitis had higher risks for uveitis events in case of etanercept and no first event of uveitis occurred during adalimumab treatments. In contrast, MTX seems to have a protective effect on the occurrence of uveitis. In 16 of 3,467 patients, the first uveitis event occurred during the observation period, 11 while taking MTX without a biologic agent (0.32/100 patient-years), two while taking etanercept monotherapy (0.19/100 patient-years), and three while taking etanercept and MTX in combination (0.09/100 patient-years) [38]. A previous open-label study showed that MTX is an effective treatment for uveitis; approximately 60–70% of patients responded to the treatment and had a longer remission rate while taking MTX. Therefore, MTX seems to exert protective effects against the recurrence of uveitis [39, 40]. In our study, new-onset uveitis occurred in five patients (1.5/100 patient-years) receiving etanercept. Compared with previous studies, this study shows a relatively high incidence. Three of the patients were females and FANA (+), which accounted for 13% of total FANA (+) patients included in our study. Their characteristics did agree with the known risk factors. Foeldvari et al also found that MTX seems to have a protection against uveitis recurrence, as monotherapy and in combination with etanercept [38]. In the present study, we could not observe the protection effect of MTX on uveitis recurrence because all patients reported occurrence of uveitis for the first time. The prevalence of each JIA category varies between different races and oligoarthritis was relatively less frequent in Asia [41, 42]. According to the studies in Korea and Japan, the polyarthritis was the most prevalent and the oligoarthritis was a relatively low incidence compared to Western countries [43, 44]. The disease outcomes and JIA-related uveitis could probably be affected by demographic distinction between Caucasians and Asians. The high incidence of uveitis reported in our study requires caution in interpreting even when considering ethnic differences. This is because it is possible to include patients who have not been diagnosed with uveitis before the start of etanercept. Saurenmann et al reported a mean of 6.9 years (range, 0 to 23.2) before the development of uveitis, and our patients showed a mean of 5.0 years (range, 1.7 to 8.0) from the beginning of etanercept and 8.0 years (range, 2.8 to 12.5) from the diagnosis of JIA[36]. Therefore, we should keep in mind that it is still not possible rule out the effects of disease.

Although this study is performed by retrospective exploration, pharmacy-based surveillance of ADEs demonstrated a significantly higher reporting rate and sensitivity for detecting ADEs than the medical chart review alone did [45]. Clearly, our study has some limitations that support the possibility of underreporting. First, surveillance is dependent on chart audit for ADE detection. Consequently, ADEs that may be suspected but not documented in patient medical records are not detected and reported by the chart audit system. Second, the lag time between ADE detection and hospital visits existed owing to patient recall error. Third, we evaluated ADEs under ongoing therapy and did not included a three-month risk window of BAs. It means that we could not detect ADE within 3 months after biologics discontinued. Therefore, we presume that ADEs detected in the medical records are not consistent with those seen in patients. It is important to note that careful interpretation is needed when comparing our studies with other register studies. Lastly, this was a single-center study at an academic hospital with a medically complex patient population. Replication of our results at other institutions and in other patient populations is necessary to enhance the generalizability of our findings. In order to have sufficient data to evaluate a possible link between JIA, treatment and malignancy, a rigorous surveillance system is required with a very large sample size and an adequate follow-up period.

In our long-term retrospective experience of JIA patients treated with etanercept, unexpected ADEs were not apparent in controlled trials required for registration. There were no cases of tuberculosis, other opportunistic infections, malignancies, or deaths described in other recent studies. However, in case of etanercept, infection including URIs was the most common ADE and concomitant corticosteroids contributed to the risk of infections. ISRs were also frequent and affected by ingredient difference in a single use prefilled syringe. In conclusion, etanercept appear well-tolerated overall and its risk-benefit profile strongly favors benefit. Risks can be largely prevented by appropriate screening and careful monitoring. To gain further knowledge about risk profiles, national and international collaboration for the accumulation of long-term data should be encouraged in Korea.
